# Hemophagocytic Lymphohistiocytosis: A Case Series From a Tertiary State Hospital in Malaysia and a Review of Current Literature

**DOI:** 10.7759/cureus.61636

**Published:** 2024-06-04

**Authors:** Jing Yi Khaw, Wee Fu Gan, Hwee Cheng Chong, Ngee Siang Lau, Wan Aswani Wan Yusof

**Affiliations:** 1 Medical Department, Hospital Melaka, Melaka, MYS; 2 Infectious Diseases Department, Hospital Melaka, Melaka, MYS; 3 Hematology Department, Hospital Ampang, Ampang, MYS; 4 Pathology and Laboratory Medicine Department, Hospital Melaka, Melaka, MYS

**Keywords:** lymphoma, adult onset still's disease, hscore, bmat, hlh

## Abstract

Introduction: Hemophagocytic lymphohistiocytosis (HLH) is a lethal emergency. Delays in diagnosis and treatment are detrimental to the health of patients. Classical clinical manifestations of HLH include fever, cytopenia, liver dysfunction, central nervous system involvement, and coagulopathy.

Methods: We report seven cases of secondary HLH in adults diagnosed from a total of 1200 bone marrow aspiration and trephine biopsy (BMAT) examinations in our center, with various presentations and underlying triggers including infection, malignancy, and autoimmune disease.

Results: HLH can present with non-specific signs and symptoms.

Conclusion: Early recognition of HLH is crucial to enable the commencement of therapy as early as possible to prevent mortality resulting from multi-organ failure.

## Introduction

Hemophagocytic lymphohistiocytosis (HLH) is a severe hyperinflammatory syndrome induced by aberrantly activated macrophages and cytotoxic T cells [1]. HLH is classified into primary and secondary forms. The primary (genetic) form, caused by mutations affecting lymphocyte cytotoxicity and immune regulation, is most prevalent among children, while the secondary (acquired) form is most common among adults. Secondary HLH is notably triggered by infection, malignancy, and autoimmune disorders [1-4]. One of the main challenges in diagnosing HLH is when the patient presents with features that are indiscernible from sepsis or multiple organ dysfunction syndrome [1].

The diagnosis of HLH is based on clinical and laboratory criteria according to the International Society of Histiocytosis in 2004 [1]. An alternative diagnostic tool is the HScore which has been validated for reactive hemophagocytic syndrome [5]. Due to a state of hyperimmune activation and a potential severe cytokine storm, patients often experience recurrent fever, cytopenia, liver dysfunction, and a sepsis-like syndrome that may instantaneously progress to terminal multiple organ failure [1].

Treatment is devised according to the HLH classification, triggering factor, and the clinical evolution of the disease. In primary HLH, etoposide and dexamethasone (as in HLH-94 or HLH-2004 protocol) are used but a potential curative therapy is hematopoietic stem cell transplantation (HSCT) [6]. On the other hand, individualized adaptation of immunosuppression with or without chemotherapy is recommended in adult patients with secondary HLH; this strategy is instituted with concurrent treatment of the underlying trigger [1]. Macrophage activation syndrome (MAS) is a form of secondary HLH that does not usually require chemotherapy in its treatment except in patients with very severe disease or central nervous system involvement [1].

## Materials and methods

**Figure 1:**
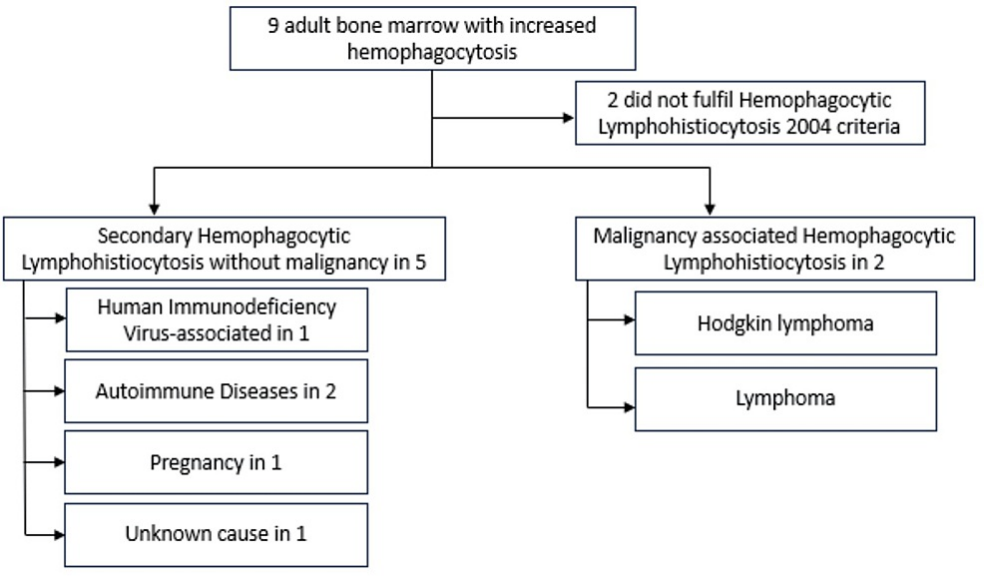
Bone marrow aspiration and trephine biopsy examinations performed in our center between 2017 and 2022 and identified cases of increased hemophagocytosis as well as the etiology

The present study is an observational, cross-sectional, and retrospective single-site study of adult patients diagnosed with secondary HLH based on the HLH-2004 criteria proposed by the Histiocyte Society in the Department of Medicine and Pathology of Hospital Melaka, Melaka, Malaysia. All adult patients above 18 years of age admitted to our medical wards with the diagnosis of HLH were included in the study. We reviewed all the reports of bone marrow aspiration and trephine biopsy (BMAT) examinations performed in our center between 2017 and 2022 and identified cases of increased hemophagocytosis as well as the etiology (Figure 1).

Data regarding patients' demographics (age and gender), clinical presentations (presence of fever, arthritis, rashes, jaundice, neurological symptoms, shock, hepatosplenomegaly), laboratory results of blood tests (full blood count, ferritin, triglycerides, lactate dehydrogenase, and fibrinogen), BMAT findings, therapy administered, and clinical outcomes (recovered or died) were reviewed. All the information was retrieved manually from patient medical records.

We further reviewed computerized hospital records using "hemophagocytic lymphohistiocytosis" as the search term in the listed patient diagnoses on discharge for any additional cases (those not detected from BMAT) of HLH. However, there was none.

## Results

Nine cases of HLH were diagnosed from an analysis of 1200 bone marrow aspirate and trephine performed between January 2017 and December 2022 (Table 1). Of these, two were in the pediatric age group.

**Table 1:** Nine cases of HLH were diagnosed from an analysis of 1200 BMAT performed between January 2017 and December 2022, of these, two were in the pediatric age group BMAT: bone marrow aspiration and trephine biopsy; HLH: hemophagocytic lymphohistiocytosis

Year	Number of bone marrow aspiration and trephine biopsy performed	Number of bone marrow aspiration and trephine biopsy with increased hemophagocytosis reported	Fulfilled hemophagocytic lymphohistiocytosis criteria
2017	185	3	1
2018	180	1	1
2019	218	1	1
2020	243	2	2
2021	214	0	0
2022	160	4	4
Total	1200	11	9

Patients’ demographics, clinical features, laboratory blood results, bone marrow findings, the HScore, treatment history, and outcomes are summarized (Table 2).

**Table 2:** Patients’ demographics, clinical features, laboratory blood results, bone marrow findings, the HScore, treatment history, and outcomes

Patient	A	B	C	D	E	F	G
Age (years)	47	43	31	18	25	32	52
Gender	Female	Female	Female	Female	Male	Male	Female
Fever	Yes	Yes	Yes	Yes	Yes	Yes	Yes
Arthritis	Yes	No	No	No	No	No	No
Rashes	No	No	No	No	No	No	Yes
Jaundice	No	No	No	No	No	No	No
Neurologic	Absent	Absent	Absent	Absent	Absent	Present	Absent
Shock	No	No	Yes	No	No	No	Yes
Hepatomegaly	Yes	Yes	Yes	No	Yes	Yes	Yes
Splenomegaly	Yes	No	Yes	No	Yes	Yes	No
Etiology	Adult-onset Still's disease	Systemic lupus erythematosus	Pregnancy	Idiopathic	Hodgkin's lymphoma	Retroviral disease	Lymphoma
Cytopenia	Anemia	Anemia	Pancytopenia	Bicytopenia	Pancytopenia	Pancytopenia	Bicytopenia
Triglyceride (mmol/L)	7.02	2.46	6.11	1.61	3.46	3.70	4.30
Fibrinogen (g/L)	2.38	1.58	5.69	3.32	Not sent	4.23	0.68
Ferritin (μg/L)	>36300	>36300	4212.8	2425.2	23912.8	7460.9	>36300
LDH (U/I)	2655	458	860	969	Not sent	155	1200
Hemophagocytosis on bone marrow aspiration and trephine biopsy	Yes	Yes	Yes	Yes	Yes	Yes	Yes
Positive CD68	Yes	Yes	Yes	Yes	Yes	Yes	Yes
Liver dysfunction	Yes	No	Yes	Yes	No	Yes	Yes
HScore	286	235	275	158	251	269	260
Comorbidities	Hypertension	Evan’s syndrome (diagnosed in 2007, done splenectomy in 2018, systemic lupus erythematosus, bronchial asthma, hypertension, diabetes autoimmune hemolytic anemia, history of smear negative pulmonary tuberculosis in 2018, completed 9 months of anti-TB therapy, history of deep vein thrombosis	Antenatal history of anemia in pregnancy (baseline Hb of 9 g/dL) and gestational diabetes mellitus	No known medical illness	Refractory Hodgkin lymphoma with marrow involvement (Stage 4B mixed cellularity) diagnosed in 2019	No known medical illness	No known medical illness
Treatment history	Steroids	Steroids + cyclosporine	Steroids	Steroids	Steroids + chemotherapy	Steroids + antiretroviral therapy + intravenous immunoglobulin	Steroids + chemotherapy
Outcome	Recovered	Recovered	Recovered	Recovered	Died (13 days from diagnosis to death)	Died (13 days from diagnosis to death)	Died (5 days from diagnosis to death)

**Figure 2:**
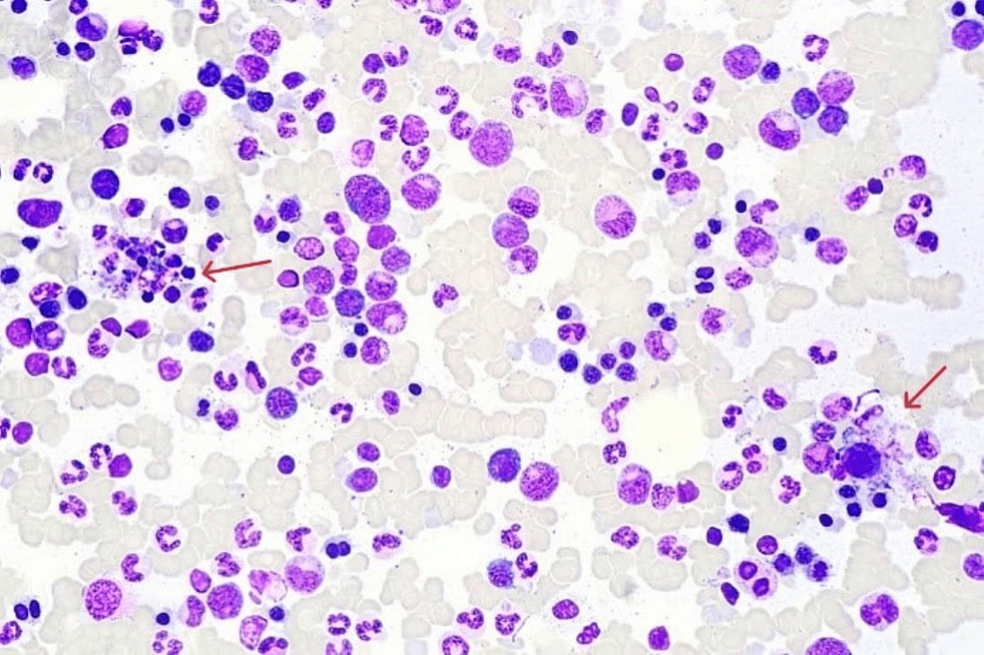
Bone marrow aspiration biopsy. Red arrow pointing towards hemophagocytic activity on bone marrow aspiration biopsy

**Figure 3:**
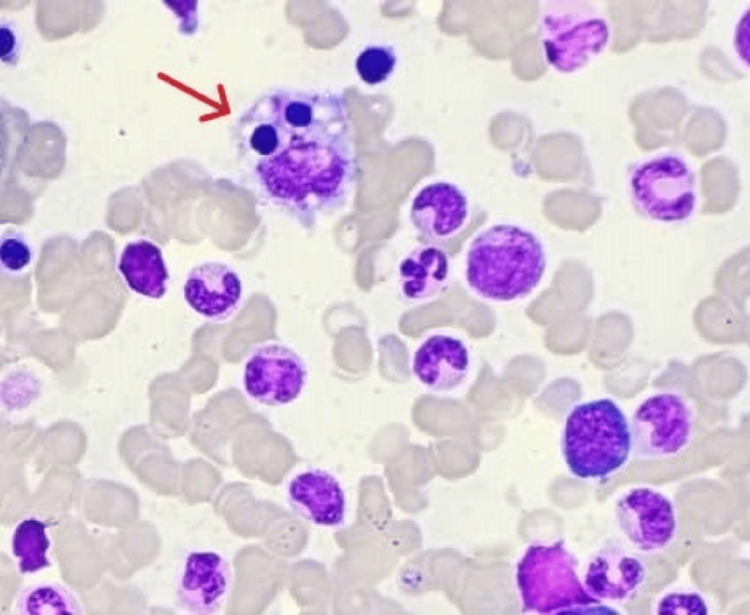
Bone marrow aspiration biopsy. Red arrow pointing towards hemophagocytic activity on bone marrow aspiration biopsy

In our case series, the age of the adult patients ranged from 18 years to 52 years. There were five females and two males. All seven patients presented with fever. Four had hepatosplenomegaly clinical and radiologically at presentation. Only one had neurological symptoms which manifested as headache in a patient with human immunodeficiency virus infection. All seven patients had cytopenia affecting one to three cell lineages - two with anemia, two with bicytopenia, and three had pancytopenia. Further blood testing showed all seven adult HLH patients had hyperferritinemia. Hypertriglyceridemia was present in five patients and hypofibrinogenemia in one. On the other hand, five patients had associated liver dysfunction; however, there was no biochemical raised bilirubin rendering jaundice state in our patients. Two patients had raised LDH. All bone marrow smears in these patients showed an increased number of histiocytes with features of hemophagocytosis (Figures 2, 3). All patients showed positive CD68 staining for increased phagocytosis.

Three patients in our case series, which include both lymphoma cases, did not respond to treatment instituted using the HLH-2004 protocol after diagnosis was established based on histological evidence in the bone marrow examinations, and they eventually succumbed to classical Hodgkin lymphoma with mixed cellularity, T cell lymphoma, and retroviral disease. Meanwhile, the other four of our patients responded to our treatment and survived.

## Discussion

Due to an overlap of symptoms and the lack of specificity of laboratory findings, diagnosis of HLH remains a challenge [7]. Treatment is often lingered as a result of the clinician’s failure to recognize this disease. Typically, HLH progresses rapidly and frequently results in death from hemorrhage, multi-system organ failure, and infections. A high index of medical suspicion is essential to prompt early treatment. Survival from HLH requires early recognition of this syndrome and aggressive treatment of the underlying cause.

Clinical and laboratory diagnostic criteria for the diagnosis of HLH are set according to Henter’s modified criteria (Table 3) [8].

**Table 3:** HLH-2004 diagnostic criteria Reference: Adapted from Henter et al., 2007 [7] HLH: Hemophagocytic lymphohistiocytosis

Hemophagocytic lymphohistiocytosis can be diagnosed if criterion 1 or 2 is met.
1. Molecular diagnosis consistent with hemophagocytic lymphohistiocytosis
2. Diagnostic criteria for hemophagocytic lymphohistiocytosis met (5 of the 8 criteria below)
Fever
Splenomegaly
Cytopenias (affecting >2 of 3 lineages in the peripheral blood
Hemoglobin <90 g/L (hemoglobin <100 g/L in infants <4 week)
Platelets <100 x 10^9^/L
Neutrophils <1.0 x 10^9^/L
Hypertriglyceridemia and/or hypofibrinogenemia
Fasting triglycerides >3.0 mmol/L (i.e., >265 mg/dL)
Fibrinogen <1.5 g/L
Hemophagocytosis in bone marrow or spleen or lymph nodes. No evidence of malignancy.
Low or no natural killer cell activity (according to local laboratory reference)
Ferritin >500 μg/L
sCD25 (i.e., soluble IL-2 receptor) >2400 U/mL

Patients must fulfill molecular diagnosis for HLH, or 5 out of the 8 criteria for diagnosis: fever, splenomegaly, hyperferritinemia, hypertriglyceridemia and/or hypofibrinogenemia, cytopenias affecting > 2 lineages, hemophagocytosis (in bone marrow, spleen or lymph node), impaired natural killer (NK) cell function and elevated soluble CD25 (sCD25). Among the laboratory tests, several trials have described ferritin as an essential marker of disease activity, response to therapy, and prognosis [8-10]. All our patients had very high ferritin levels. Almost all of them manifested liver dysfunction and increased triglyceride levels. Due to limited resources, sCD25 and NK cell activity was not performed in our cohort.

An alternative diagnostic tool is the HScore which has been validated for reactive hemophagocytic syndrome (Table 4) [5].

**Table 4:** HScore ^*^HIV positive or receiving long-term immunosuppressive therapy (ie, glucocorticoids, cyclosporin A, azathioprine). ^¥^Defined as a hemoglobin level of 9.2 g/L and/or a leucocyte count <5 x 109/L and/or a platelet count <110 x 109/L. Reference: Adapted from Fardet et al., 2014 [10]

Parameter	Criteria for scoring
Known underlying immunosuppression^*^	0 (no) or 18 (yes)
Temperature (^o^C)	0 (<38.4), 33 (38.4-39.4), or 49 (>39.4)
Organomegaly	0 (no), 23 (hepatomegaly or splenomegaly), or 38 (hepatomegaly and splenomegaly
No. of cytopenias^¥^	0 (1 lineage), 24 (2 lineages), or 34 (3 lineages)
Ferritin (μg/L)	0 (<2000), 35 (2000-6000), or 50 (>6000)
Triglyceride (mmol/L)	0 (<1.5), 44 (1.5-4), or 64 (>4)
Fibrinogen (g/L)	0 (>2.5) or 30 (<2.5)
Aspartate aminotransferase (U/L)	0 (<30) or 19 (>30)
Hemophagocytosis on bone marrow aspirate	0 (no) or 35 (yes)

On the contrary, BMAT with hemophagocytosis finding has a sensitivity of 60% despite it being more commonly used to diagnose HLH [11]. Hence, a normal bone marrow biopsy should not exclude or delay the initiation of treatment when there is a strong clinical suspicion of HLH [11]. Unfortunately, our center has no access to laboratory facilities to perform more specialized immunological tests and genetic assays, like functional measurement of NK cells and levels of soluble CD25 (soluble interleukin-2 receptor).

The mainstay of treatment is to reduce hyperinflammation which is responsible for fatal manifestations caused by HLH and also to treat the underlying etiology to halt the progression of the disease [12]. The first line of management includes corticosteroids and adequate control of triggering factors, to prevent potential deaths from sepsis, disseminated intravascular coagulation (DIC), and multi-organ failure [12].

**Figure 4:**
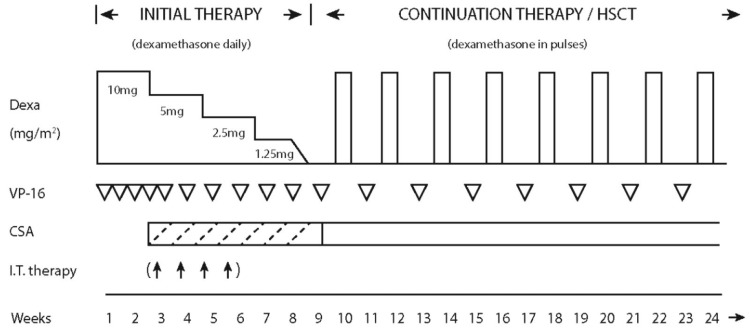
HLH-2004 treatment protocol Reference: Adapted from Henter et al., 2007 [7] CSA: cyclosporine A; HSCT: hematopoietic stem cell transplantation

The HLH-2004 treatment protocol (Figure 4) is the mainstay therapy in newborns, infants, and children up to 18 years of age, in whom genetic causes are the dominant factors.

It comprises eight weeks of initial therapy followed by a continuation phase, in which both include the use of etoposide and dexamethasone [8,13]. Initial therapy was intensified by early administration of cyclosporine A (CSA) to increase immunosuppression without inducing additional myelotoxicity. HSCT is recommended to be performed as soon as an acceptable donor is available.

Four patients recovered as HLH was suspected early and treatment was promptly initiated. Unfortunately, three presented with sepsis and died of severe infections complicating HLH. In retrospect, our patients with severe disease and life-threatening manifestations might have had a more favorable outcome if a more aggressive approach was adopted with early use of etoposide, especially after an unsatisfactory response to corticosteroids and treatment of its precipitating cause [14].

Our case series is a small review of seven patients as we were only able to identify patients with increased hemophagocytosis from the analysis of BMAT. Hence, we were unable to derive any meaningful conclusions. We are aware there is a high possibility that HLH might have been missed or underdiagnosed in our center due to the lack of awareness among the clinicians. This was similarly described by another author [15]. To the best of our knowledge, this is the first case series on secondary HLH from a tertiary state hospital.

## Conclusions

HLH is a lethal emergency. Delays in diagnosis and treatment of this condition may jeopardize the lives of patients. HLH often presents with non-specific symptoms and signs that are similarly found in disorders that trigger HLH such as lymphoma or sepsis. Common presentations of HLH are fever, neurological symptoms, coagulopathy, cytopenia (in particular, thrombocytopenia), and liver dysfunction. The mainstay of management in adult patients is steroids with concurrent treatment of the underlying etiology. Escalation of therapy to include etoposide can be considered in patients who do not respond well to corticosteroids. In summary, clinicians should have a high index of suspicion when managing patients who may not always present with typical features of HLH. We should promptly investigate for clinical markers of this syndrome in the early phase of illness and initiate therapy as soon as possible, to prevent multi-organ failure which leads to high morbidity and mortality.
